# The Effects of a 10-Week Aerobic and Unilateral Lower Extremity Resistance Training Program on Amplitude and Nerve Conduction Velocity of Sensory and Motor Nerves in Diabetic Patients with Neuropathy

**DOI:** 10.5114/jhk/161610

**Published:** 2023-04-20

**Authors:** Sharif Beigi, Fatemeh Shabkhiz, Mohammadreza Kordi, Bahram Haghi-Ashtiani, Nahid Hashemi-Madani, Piotr Zmijewski

**Affiliations:** 1 Department of Exercise Physiology, Sport Sciences and Health Faculty, University of Tehran, Tehran, Iran.; 2 Department of Neurology, Firoozgar Hospital, Iran University of Medical Science, Tehran, Iran.; 3 Endocrinology and Metabolism Research Center, Institute of Endocrinology and Metabolism, Iran University of Medical Science, Iran.; 4 Jozef Pilsudski University of Physical Education in Warsaw, Warsaw, Poland.

**Keywords:** complex training, nerve conduction, glycaemic control, diabetic neuropathy

## Abstract

This study aimed to investigate the effects of 10-week aerobic and unilateral lower extremity resistance training on nerve conduction velocity and amplitude of sensory and motor nerves in diabetic patients with neuropathy. This clinical trial was conducted on twenty women and men (aged 30–60 years old) with diabetic neuropathy. Participants were randomly assigned to one of the two groups: an exercise group (EG; n = 10) and a control group (CG; n = 10). The EG performed a 10-week programme with one session of aerobic exercises (40% to 70% of HR reserve), supplemented with one session of specific lower extremity resistance exercises (60–90 min/day) on the same day for four days per week. The CG subjects performed their regular daily activities. The nerve conduction velocity, amplitude of sensory and motor nerves and glycosylated haemoglobin A1c were measured before and after the intervention. The repeated-measures ANOVA showed a significant increase in the conduction velocity of the sural sensory nerve as well as the peroneal motor nerve (p < 0.01, p < 0.01). The changes in the conduction velocity of the tibial nerve were similar when compared to the control group (p > 0.05). A significantly greater decrease in glycosylated haemoglobin was also observed in the EG group (p < 0.01). Performing 10 weeks of aerobic and specific unilateral lower extremity exercises can improve the function of sensory and motor nerves and improve symptoms in diabetic patients with neuropathy. Given the limited studies in this area, the exact mechanisms of this performance improvement need further examination.

## Introduction

Diabetes mellitus is a metabolic disorder characterized by abnormal carbohydrate metabolism (Agarwal et al., 2018) caused by a deficiency in insulin secretion, insulin function, or both (Kiani et al., 2018). According to the International Diabetes Federation, in 2019, 462 million people (9.3% of the world's population) and 5.4 million Iranians (9.4% of Iran’s population) had diabetes ( Abdi et al., 2019; Ahmad et al., 2017; Arazi et al., 2020; Cade, 2008; Guariguata et al., 2014; Said, 2007). Diabetes is associated with several other complications during its clinical course, including small vascular disorders (retinopathy, nephropathy and neuropathy) and large vascular disorders (e.g., atherosclerosis) (Boulton et al., 1998; Dyck et al., 1993). Diabetic peripheral neuropathy (DPN) is a major consequence of diabetes, affecting between 30–50% of diabetic people (Callaghan et al., 2012; Deshpande et al., 2008). DPN is the progressive destruction of peripheral nerves, especially in the lower extremities (Kumar et al., 1994), which affects the sensory, motor and automatic components of the peripheral nerves. DPN is a known complication of microvascular disease in diabetic patients (Callaghan et al., 2012). Distal symmetrical polyneuropathy (DSPN) is the most common type of DPN diagnosed, characterized by dysfunction of sensory and motor nerves in a time-dependent manner (Gore et al., 2005; Said, 2007). Approximately 20% of DSPN patients suffer from severe pain, and DSPN is a major cause of disability and decreased quality of life (Wu et al., 2007). The most serious complication of DSPN is a foot ulcer, which can eventually lead to amputation of a limb. DSPN increases the risk of foot ulcers by up to sevenfold and contributes to more than 60% of lower-limb amputations in diabetic patients (Moghtaderi et al., 2006). Early diagnosis of neuropathy may be a means of identifying patients at high risk for lower extremity complications. At the same time, it allows for early intervention and treatment, which leads to a better prognosis for these patients. In the presence of clinical signs of neuropathy such as anaesthesia and numbness, the neurological examination is a standard and sensitive method to assess changes in these patients.

Nerve conduction studies (NCS) are the current objective measure and provide an accurate, valid, non-invasive tool for assessing diabetic neuropathy (Agarwal et al., 2018; Dyck et al., 2011). In NCS, conduction velocity and amplitude of action potential were selected as relevant variables (Van Sloten et al., 2011). The presence of DPN in adults reduces the levels of physical activity measured by the number of steps per day (Boulton et al., 2008; Maluf and Mueller, 2003). To date, no definitive treatment has been described for diabetic peripheral neuropathy, and most treatments focus on pain relief strategies. It is well established that diabetes and its associated complications can be prevented by fine-tuning blood glucose through diet, exercise, and medication (Kluding et al., 2017; Sigal et al., 2007; Nemati et al., 2021). Recently, several large-scale clinical trials have shown that aerobic exercise improves physical fitness, glycaemic control, and insulin sensitivity in diabetics (Pashaei et al., 2013; Poirier et al., 2000). Nerve damage from diabetes is complex and involves many mechanisms, but nerve damage from diabetes may be rooted in metabolic or vascular disorders (Gholami et al., 2018). For example, Agarwal et al. (2018) found that nerve conduction velocities in the sensory and motor nerves are lower in people with higher HbA1c levels. Also, the conduction velocity and amplitude of the sural nerve worsen with the increasing duration of the disease (Agarwal et al., 2018). In addition, due to the role of vascular factors in the development of peripheral neuropathy, improving the blood flow to the limbs can enhance nerve conduction velocity in these patients (Kluding et al., 2012). Kiani et al. (2018) found that performing six weeks of aerobic exercise significantly increased the nerve conduction velocity in the median, ulnar, peroneal and tibial nerves of diabetic neuropathy. In that study, the amplitude of action potential in these nerves significantly increased compared to the control group (Kiani et al., 2018). Therefore, regular exercise due to metabolic and vascular effects may be effective in preventing the progression of peripheral neuropathy. For example, Gholami et al. (2018) in their study examined the effects of 12 weeks of aerobic exercise on nerve conduction velocity and blood sugar in people with diabetic neuropathy and found that aerobic exercise significantly increased nerve conduction velocity and amplitude of action potential and decreased blood sugar (Gholami et al., 2018). In contrast, Kluding et al. (2012) in an experimental study examined the effects of aerobic-resistance training on symptoms, neuronal function and cutaneous nerves in patients with diabetic peripheral neuropathy and they reported that performing 10 weeks of aerobic-resistance training did not have significant effects on nerve conduction velocity and amplitude of tibial, peroneal, and sural nerves (Kluding et al., 2012). Nikokhaslat et al. (2019) examined men with neuropathy, performing 12 weeks of resistance training with 3 sessions per week, and found significantly increased conduction velocity and amplitude of peroneal, tibial, and sural nerve. In contrast, Stubb et al. (2019) found that performing 24 weeks of exercise could not alter the electro-diagnostic responses of sensory and motor nerves, regardless of the type of exercise. A sedentary lifestyle is considered one of the major risk factors for diabetes and its consequences (Sudeck and Höner, 2011; Toledo et al., 2007). Only few studies have focused on the effects of exercise on peripheral neuropathy. This may be due to researchers' concerns about possible injuries from weight-bearing exercises in this group. Recently, however, it has been reported that patients with peripheral neuropathy can safely participate in relatively intense aerobic and resistance exercises (Stubbs et al., 2019).

Aerobic exercise can also improve cardiorespiratory fitness, and control blood sugar levels, and HbA1c in type 2 diabetics (Andrew et al., 2004). Resistance training can improve muscle strength and performance (Liao et al., 2022) and control blood sugar and HbA1c levels (Castaneda et al., 2002.; Pan et al., 2018). Aerobic and resistance training (combined) has been recommended by the European Heart Association (Colberg et al., 2010), the American Sports Medicine Association (Hordern et al., 2012), the Belgian Physical Therapy Association and the Exercise Association of Australia (Brehm et al., 2009). It was suggested that sedentary people wishing to attain the health and fitness benefits of exercise can choose from a wide range of potential exercise as so long as the effort is high (Fisher and Steele, 2014).

Pan et al. (2018( showed that performing combined training can be more effective in reducing HbA1c and consequences of diabetes, compared to aerobic and resistance training alone, but further research in this area is recommended. Very few studies have examined the effects of combined exercise on peripheral nerve function in patients with diabetic neuropathy. Based on previous studies, aerobic exercises (Dixit et al., 2014; Gholami et al., 2018) and unilateral lower extremity exercises (Cooper et al., 2016; Nadi et al., 2019) can potentially be effective through independent mechanisms, and the combination of these exercises will probably increase this effect. Therefore, the aim of this study was to investigate the effects of aerobic and unilateral lower extremity exercises on nerve conduction velocity and amplitude in sensory and motor nerves.

## Methods

### Participants

**Figure 1:**
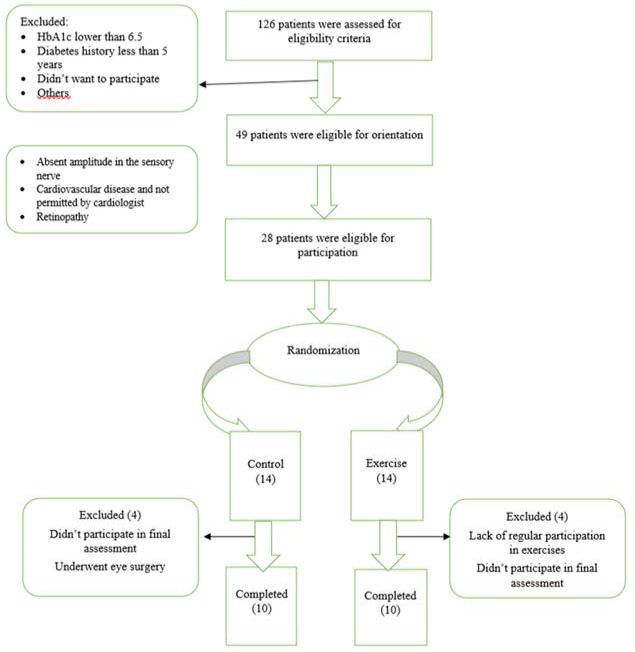
Participants’ recruitment flow chart.

The research was a quasi-experimental two-group study (experimental and control) with pre-/post-test measurements. Twenty-eight participants were recruited for this study among patients referred to the Research Institute of Endocrinology and Metabolism of the Iran University of Medical Sciences (men and women) with diagnosed type 2 diabetes with peripheral neuropathy in the age range of 30 to 60 years. The participant recruitment flow chart is shown in Figure 1. Twenty individuals completed the intervention (11 women and 9 men). They had not undergone any regular resistance training within the past 1 year and had no previous musculoskeletal injuries of the extremities before the study. They were provided with a detailed explanation of the study protocol before participation and signed an informed consent form. The study was approved by the Ethics Committee of the University of Tehran and registered in the Iranian Clinical Trial Registration Canter (IR.UT.SPORT.REC.1400.014).

Inclusion criteria were: the diagnosis of type 2 diabetes for more than 5 years, glycosylated haemoglobin between 6.6% and 12%, lack of regular exercise, lack of specific medical conditions such as a history of heart attack and stroke, no medical contradictions of exercise, report of diabetic peripheral neuropathy in medical records, presence of clinical signs of diabetic peripheral neuropathy according to the Michigan questionnaire.

### Research Procedures

After the initial examination, 28 individuals were introduced to the training and examination protocol. Initially, a clinical examination of the subjects' feet was performed by a technician to diagnose the loss of protective sensation (LOPS) in the foot clinic of the Endocrine and Metabolism Research Institute of the Iran University of Medical Sciences, which included: a 10 g monofilament, 128 Hz tuning fork, pinprick sensation, ankle reflex, and foot temperature (cold-warm test). Nerve conduction examinations were then performed by a neurologist on the right legs of the subjects.

Participants were randomly assigned to exercise (EG, n = 14), and control (CG, n = 14) groups. CG subjects were asked to maintain their normal diet and daily activities as well as medication intake during the 10-week experiment and to notify the researcher in the event of any changes. The EG, in addition to maintaining a normal diet, performed exercises 4 times per week in the morning during the training days in the gym. The intensity and duration of exercises were according to the training protocol (Table 1). Each training session started with a 10-min warm-up, including walking and stretching exercises, followed by aerobic exercises on the treadmill and then lower extremity exercises based on the previously applied protocol (American Diabetes Association, 2008; Poirier et al., 2000; Stubbs et al., 2019) and at the end of each session, a 10-min cool down was performed. The intensity of aerobic exercise for each individual was determined using the Karvonen equation (Gholami et al., 2018). The subjects' heart rate was monitored by a Polar heart rate monitor during each session. Also, in order to prevent possible consequences and hypoglycaemia, the subjects' blood sugar was monitored before and after training using a glucometer, and if the blood sugar levels were inappropriate, the training session was cancelled.

**Table 1:** Exercise training protocol.

Aerobic exercises				Unilateral exercises	Set x repetitions
Weeks	Time [min]	Intensity %HRR	Sessions per weeks		
1st and 2nd	20	40–55%	4	1. Dorsi flexion side-lying	3 x 5
3rd and 4th	25	50–55%	4	2. Plantar flexion on gym ball	3 x 5
5th and 6th	30	55–60%	4	3. Raise the heel for 20 s	1 x 5
7th and 8th	35	60–65%	4	4. Stand on one foot for 15 s	1 x 5
9th and 10th	40	65–70%	4	5. Unipedal wobble board 6. Foot inversion 7. Foot eversion 8. Toe extension on the step 9. Toe flexion on the step 10. Tennis ball rolling 11. Ankle rotation	1 x 3 3 x 5 3 x 5 3 x 5 3 x 5 1 x 5 3 x 5

### Blood Sampling

Twenty-four hours before the first training session and 48 hours after the last one, participants attended the laboratory for blood collection under the fasting condition. Participants were asked to refrain from any exercise for 48 hours before blood sampling. A 10 ml blood sample was taken from the cubital vein. Blood samples were poured into separate tubes immediately after blood sampling. In each session, glycosylated haemoglobin levels were measured immediately after blood sampling. The rest of the blood samples were centrifuged to isolate the serum.

### Electrophysiological Examination

Nerve conduction velocity and action potential amplitude examinations were performed with a Viking Quest (Nicolet VIASYS Healthcare, USA). Since patients in this study had symmetrical distal polyneuropathy in the lower extremities, examinations were performed on a sensory nerve (sural) and two motor nerves (peroneal and tibial) in the right legs of all subjects. For the tibial motor nerve examination, the active recorder (G1) was placed on the abductor hallucis brevis belly and reference (G2) on the first metatarsophalangeal, and the peroneal motor nerve was examined by placing G1 on the extensor digitorum brevis (EDB) belly and G2 on the 5^th^ metatarsophalangeal. In the sural sensory examination, G1 and G2 were put on the sural nerve in the lateral ankle, by a 3 to 4-cm distance. Recording filters were 20 to 2 kHz. Stimulation duration was 0.2 millisecond, 20 to 50 milli-Amper for the motor nerve and 15 to 30 milli-Ampers for the sensory nerve examination. Stimulators were placed on the distal tibial and peroneal of the ankle and proximal to the knee. The amplitude of motor and sensory nerve action potential were recorded in millivolt and microvolt, respectively. Compound motor action potential amplitudes (APA) and motor nerve conduction velocities were determined in accordance with Dixit et al. (2014).

### Nerve Conduction Velocity

The distance between the two points of nerve stimulation was measured (as L) in mm and the conduction velocity of the stimulus along the nerve was calculated by the formula V = L / (t1 − t2), where t1 is the time delay in ms between the point of nerve stimulation at the upper point of proximal stimulation, and t2 is the time calculated for the lower point or the distal stimulation. For the motor nerves of the tibia and peroneal, the nerve conduction velocity was obtained by dividing the distance between the stimulation point and recording the latency between the two points.

### Diet During Study

To match the diet in the days before the blood draw, participants were given 24-hour food reminders in the first session. They recorded the diet until the day before the first blood draw and questioned participants on the day before the final blood draw session. Participants followed the same diet. The caloric intake was calculated using a 24-hour food recall questionnaire in the first week and the last week before the end of the study. Participants' medication intake was recorded during the study based on individual reports and adjusted by an endocrinologist.

### Exercise Protocol

At first, the training group performed aerobic exercises and then unilateral lower extremity specific exercises (Nadi et al., 2019) for the lower limbs, which included exercises that involved the relevant nerves (American Diabetes Association, 2008; Poirier et al., 2000; Stubbs et al., 2019). The exercises were performed according to Table 1. These exercises were performed under the direct supervision of an exercise physiologist. Each session began with 10 minutes of warm-up exercises, including walking and stretching, and then participants performed aerobic exercises on the treadmill followed by specific unilateral exercises for the right leg. The intensity of aerobic exercise was maintained at 40 to 70% of the heart rate reserve, and was calculated using the Karvonen equation. The exercise protocol had been previously presented by Beigi et al. (2022).

### Statistical Analysis

Data were analysed using SPSS for Windows version 26 (United States, Armonk, NY: IBM Corp). of data was assessed for normality using the Shapiro-Wilk test. The data were normally distributed and therefore analysed using parametric methods. Repeated-measures analysis of variance was applied to analyse the changes in time, with significant time × group interaction showing significant dependence of the change on group. The paired *t*-test was also used to compare the pre-test (week 0) and post-test (week 10) values of nerve conduction velocity, amplitude and glycosylated haemoglobin.

## Results

Excellent adherence was obtained in our study and participants (average attendance = 95%) of the supervised exercise sessions. No serious unanticipated adverse events occurred during testing or intervention.

The increases in the nerve conduction velocity in the sural sensory (F = 6.83, *p* = 0.018) and peroneal motor (F = 9.84, *p* = 0.006) nerves were significantly greater when compared to changes in the control group (Table 2). For the tibial motor nerve, a time×group interaction effect was not found (F = 4.09, *p* = 0.058). There were no significant changes in the amplitude of the sural sensory nerve, peroneal and tibial motor nerves following exercise (Table 2). The time×group interaction for HbA1c demonstrated that exercise led to a significant decrease in concentration when compared to initial values, but only in the experimental group (F = 6.73, *p* = 0.018).

**Table 2:** Change in outcome measures before and after the exercise intervention period between groups.

Group	Experimental (n = 10)		Control (n = 10)		
	Mean ± SD		Mean ± SD		
Variable	pre	post	pre	post	Time x group interaction effect
Sural NCV [m/s]	35.91 ± 5.14	38.65 ± 6.61^*^	37.40 ± 5.53	36.54 ± 4.10	**
Peroneal NCV [m/s]	40.14 ± 4.49	43.39 ± 3.89 ^*^	41.6 ± 5.01	41.74 ± 3.98	**
Tibial NCV [m/s]	39.10 ± 5.89	41.82 ± 5.24^*^	40.80 ± 6.59	41.1 ± 6.57	ns
Sural APA [µV]	5.96 ± 2.3	6.99 ± 2.59^*^	6.15 ± 2.12	6.24 ± 2.01	ns
Peroneal APA [µV]	3.01 ± 1.15	3.22 ± 1.17^*^	3.13 ± 1.14	3.2 ± 1.11	ns
Tibial APA [µV]	6.35 ± 3.27	6.93 ± 3.52^*^	5.9 ± 3.78	6.03 ± 3.19	ns
HbA1c [%]	8.16 ± 1.05	7.45 ± 0.91^*^	8.17 ± 1.5	8.09 ± 1.38	**

NCV = nerve conduction velocity; APA = action potential amplitude;

* Significantly different from pre- to post-test values (at p < 0.05)

** p < 0.05

## Discussion

To the authors’ knowledge, this is the first study to investigate the effects of combined aerobic and unilateral lower extremity training on nerve conduction velocity of sensory and motor nerves in diabetic patients with neuropathy.

In the present study, 10 weeks of aerobic and unilateral lower extremity training improved nerve conduction velocity in the sural sensory nerve as well as peroneal motor nerve and significantly reduced glycosylated haemoglobin A1c in patients with diabetic neuropathy. Exercise can reduce the body's reliance on the polyol-sorbitol pathway by modulating sorbitol levels in patients with diabetic neuropathy (Dixit et al., 2014). Increased sorbitol concentration in diabetic patients has detrimental effects on Schwann cells in peripheral nerves (Dixit et al., 2014). This increase in sorbitol concentration can also reduce the endoneurial blood flow and chronic hypoxia. Increased conduction velocity of the sural sensory nerve in this study is in line with the findings of Dixit et al. (2014). They reported that performing 8 weeks of aerobic exercise with an intensity of 40 to 60% of the reserve heart rate improved the conduction velocity of the sural sensory nerve in men and women with diabetic neuropathy (Dixit et al., 2014). In contrast, Stubb et al. (2019) reported that performing 12 weeks of aerobic, resistance and combined training did not have significant effects on the function of the sural sensory nerve. Possible reasons for the different results from the present study are the number of training sessions per week, which was 3, and the intensity of exercise, set at 60 to 70% of peak oxygen uptake (Stubb et al., 2019). In contrast, in the present study, training sessions were performed 4 times per week with an intensity of 40 to 70% of the reserve heart rate. It has been well established that the effects of exercise on sensory nerves are due to the activation of afferent nerves from active muscles to the spinal cord and the activation of sensory fibres in active muscles increases during exercise (Cooper et al., 2016). Recent studies have also shown that cellular and molecular changes in sensory nerves can occur after short-term exercise (Cooper et al., 2016; Molteni et al., 2004). These effects may be due to the increased production of a number of molecular pathways, including brain-derived neurotrophic factor (BDNF), nerve growth factor (NGF), neurotrophin 3 (NT3), and synapsin 1 (SNAP1). In this study, we also observed a significant improvement in glycaemic control by measuring haemoglobin A1C levels in patients with diabetic neuropathy. Performing aerobic exercise can improve sensory nerve function by modulating metabolic and vascular pathways in patients with diabetic neuropathy (Dixit et al., 2014). In addition, hyperglycaemia increases the production of superoxide, AGES formation and activation of protein kinase C, which inactivates the production of nitric oxide as an important mechanism in endothelial dysfunction in patients with diabetic neuropathy. Exercise can have significant effects on endothelial function and vascular dilation in patients with diabetic neuropathy. Therefore, based on previous observations, it can be assumed that improving NO-derived endothelial tissue can improve nerve function in patients with neuropathy (Dixit et al., 2014). In this study, the implementation of the training protocol reduced A1c values by 8.7% compared to the initial values. These results on the reduction of glycosylated haemoglobin are consistent with the findings of Gholami et al. (2018) and Kluding et al. (2012). Kluding et al. (2012) reported in their study that 10 weeks of aerobic and resistance training in men and women with diabetic neuropathy significantly reduced glycosylated haemoglobin by 7.3%. It has been shown that performing combined aerobic-resistance training is more effective in reducing A1c and the consequences of diabetes than performing these exercises alone (Pan et al., 2018). In the present study, 10 weeks of aerobic and unilateral lower extremity training improved nerve conduction velocity in the peroneal motor nerve, while this increase in nerve conduction velocity was not significant for the tibial motor nerve compared to the control group. These findings are consistent with a study conducted by Dixit et al. (2014) where performing 8 weeks of aerobic exercise at 40 to 60% of the reserve heart rate significantly increased the nerve conduction velocity in the peroneal nerve from 42.48 m/s to 45.46 m/s. In contrast, Kluding et al. (2012) reported that following 10 weeks of aerobic-resistance training three times per week, there was no significant change in peroneal nerve conduction velocity. Endothelial dysfunction in micro- and macrovascular injury plays a key role in the onset of diabetes-related outcomes such as peripheral nerve dysfunction. Also, a decreased endoneurial blood flow and increased endoneurial vascular resistance, ischaemia and hypoxia are the main pathophysiological mechanisms related to changes in peripheral nerve function in patients with diabetic neuropathy (Nukada, 2014; Stubbs et al., 2019) and exercise can prevent, reduce or improve peripheral nerve function by enhancing vascular endothelial function and increasing the intracranial blood flow.

This study has several limitations. First, the study group was relatively small (with 4 dropouts in the exercise group) and the diet was not strictly controlled, but only advised to be routine; therefore, the results may not be generalizable to a broader population. Unfortunately, no progression was applied to unilateral exercises, and this should be incorporated into further studies. Nevertheless, this study offered some reference values for further studies on the effects of combined exercise on amplitude and nerve conduction velocity of sensory and motor nerves in this specific population.

## Conclusions

In this study, 10 weeks of aerobic exercise combined with unilateral lower extremity training improved nerve conduction velocity in the sural sensory nerve and also improved peroneal motor nerve function in diabetic men and women with neuropathy. Additionally, a significant decrease in glycosylated haemoglobin A1c was observed in patients with peripheral neuropathy. The diabetic patients with neuropathy could achieve significant physical activity-related health benefits from a 10-week aerobic and unilateral lower extremity training program.
